# Application of multi-target phytotherapeutic concept in malaria drug discovery: a systems biology approach in biomarker identification

**DOI:** 10.1186/s40364-016-0077-0

**Published:** 2016-12-13

**Authors:** Protus Arrey Tarkang, Regina Appiah-Opong, Michael F. Ofori, Lawrence S. Ayong, Alexander K. Nyarko

**Affiliations:** 1 Centre for Research on Medicinal Plants and Traditional Medicine, Institute of Medical Research and Medicinal Plants Studies (IMPM), P. O. Box 8013, Yaoundé, Cameroon; 2 Department of Clinical Pathology, Noguchi Memorial Institute for Medical Research, University of Ghana, P. O. Box LG 581, Legon, Accra Ghana; 3 Department of Immunology, Noguchi Memorial Institute for Medical Research, University of Ghana, P. O. Box LG581, Legon, Accra Ghana; 4 Malaria Research Laboratory, Centre Pasteur Cameroon, BP 1274 Yaoundé, Cameroon; 5 School of Pharmacy, University of Ghana, P.O. Box LG43, Legon, Accra Ghana

**Keywords:** Malaria, Phytotherapy, Multi-target effects, Reverse pharmacology, High throughput screening (HTS), HPLC-based anti-malarial profiling, In vivo pharmacology, Pharmacokinetics

## Abstract

There is an urgent need for new anti-malaria drugs with broad therapeutic potential and novel mode of action, for effective treatment and to overcome emerging drug resistance. Plant-derived anti-malarials remain a significant source of bioactive molecules in this regard.

The multicomponent formulation forms the basis of phytotherapy. Mechanistic reasons for the poly-pharmacological effects of plants constitute increased bioavailability, interference with cellular transport processes, activation of pro-drugs/deactivation of active compounds to inactive metabolites and action of synergistic partners at different points of the same signaling cascade. These effects are known as the multi-target concept. However, due to the intrinsic complexity of natural products-based drug discovery, there is need to rethink the approaches toward understanding their therapeutic effect.

This review discusses the multi-target phytotherapeutic concept and its application in biomarker identification using the modified reverse pharmacology - systems biology approach. Considerations include the generation of a product library, high throughput screening (HTS) techniques for efficacy and interaction assessment, High Performance Liquid Chromatography (HPLC)-based anti-malarial profiling and animal pharmacology. This approach is an integrated interdisciplinary implementation of tailored technology platforms coupled to miniaturized biological assays, to track and characterize the multi-target bioactive components of botanicals as well as identify potential biomarkers. While preserving biodiversity, this will serve as a primary step towards the development of standardized phytomedicines, as well as facilitate lead discovery for chemical prioritization and downstream clinical development.

## Background

Malaria control initiatives have led to substantial improvement in the number of saved lives in vulnerable populations, particularly in those with access to effective anti-malarial drugs [1]. In spite of this, *Plasmodium falciparum* malaria remains a major public health burden in most endemic countries, where there is lack of access to modern healthcare facilities and disease monitoring is not well defined. Most of the populations in these countries resort to the use of plant-based complementary medicine for therapy [2]. Drugs derived from natural source, especially herbs, represent a significant proportion of the pharmaceutical market, particularly in malaria therapy, as well as other infectious diseases. Examples include quinine from Cinchona species, artemisinin from *Artemisia annua* and atovaquone from *Tabebuia impetiginosa* [3, 4]. Their advantage for the development of drugs comes from the synergistic interactions of their components and their innate affinity for biological receptors [5]. The pharmacological justification of these principles could provide the basis for further development of plant-based traditional medicine as a reliable therapeutic tool.

Malaria parasite has developed resistance to many of the currently available drugs, including emerging resistance to the core artemisinin component of the artemisinin-based combination (ACT) therapies [6]. This therefore underscores the need to explore new therapeutic strategies from natural products. Although the physiopathology of malaria is not fully understood, the knowledge that its pathogenesis involves multiple factors, obliges effective therapeutic approaches to shift from the conventional “one target, one drug” to a new “multi-target, multidrug” model [7, 8]. The requirements for the development of new anti-malarials to support the current therapeutic and eradication agenda include novel modes of action with no cross resistance to current drugs, multiple-stage efficacy and anti-relapsing activity [9].

Herbal medicines are preparations that contain plant parts (singly or in combination) in a pharmaceutically processed form. Like synthetic drugs, they claim therapeutic or prophylactic properties and may exhibit poly-pharmacological effects through interactions of their constituent phytochemicals. Assumed mechanistic reasons for these interactions include increased bioavailability, interference with cellular transport processes, activation of pro-drugs/deactivation of active compounds to inactive metabolites and action of synergistic partners at different points of the same signaling cascade. This concept is known as the multi-target effect [7]. The application of this concept in drug discovery requires the comprehensive characterization of the constituent phytochemicals and their bioactivities.

The multicomponent therapy forms the basis of phytotherapy or phytomedicines, where holistic therapeutic effect result from complex positive and negative interactions between the different components [10]. However, these bioactive components, known as metabolites possess high chemo-diversity and constitute the complex biological matrices of these plants. A knowledge of their pharmacological activities is necessary to understand the basis of their therapeutic effect and this necessitates broad interdisciplinary research approaches [11, 12]. Therefore, more than one biological test system is required to measure the therapeutically useful activities, in an attempt to establish consistency in herbal drugs. Moreover, the application of appropriate technology and bioassays is imperative in the application of this concept.

In the search for new lead compounds from natural source in malaria drug discovery, the efficient tracking and early identification of active constituents in complex matrices remains the most challenging step due to the chemical properties of plant extracts and the mechanism of their bioactivity [13]. However, the conventional bioactivity-guided isolation involving several iterative steps is first and foremost inefficient and time consuming, especially when the bioactive phytochemical is an unknown compound and exists as a minor component in the extract. Secondly, if bioactivities of plant extracts are as a result of the synergistic effects of phytochemicals, testing pure phytochemicals in bioassays might not be able to fully reveal their bioactivities. Finally, it requires large amounts of material, thereby destabilizing biodiversity. Though successful in the past, this classical approach relied on several iterative steps which brought about repeated isolation of the same compounds and it could not keep pace with the fast turn around and tight deadline of high throughput screening (HTS)-based programmes [14]. Moreover, in vitro efficacy assays for these bioactive compounds are usually target-driven and do not allow the detection of truly novel targets or identification of the complex summary effects due to the interactions of different constituents. This has brought about renewed interests in holistic in vivo test systems, which are more suitable to unravel synergism or identify pro-drugs which are converted metabolically to bioactive substances. To tackle these challenges, new research strategies employ tools such as metabolomics, a key in systems biology, for the diversity-oriented screening of herbal medicines. Besides, the normal pharmacological read-outs in malaria infection such as multiple-stage-antiplasmodial, anti-inflammatory, antipyretic, antioxidant and antinociceptive effects, there is the possibility of correlating these measurements in a system biological approach with analyzed metabolomics data [15, 16].

Metabolomics, is a key “omics” technology in systems biology. It is the comprehensive qualitative and quantitative analysis of the bioactive molecules in a cell, organ or organism at a specific point in time. It is a highly versatile strategy to accelerate deconvolution of active extracts, in which a variety of detection methods are adopted for metabolite analysis including electrochemical array, infrared spectroscopy, mass spectrometry (MS) and nuclear magnetic resonance (NMR). Amongst the available platforms, the hyphenation of HPLC-MS, coupled to NMR spectroscopy is the most widely used technology due to its ability to detect and separate a diverse range of molecules with high sensitivity [17].

**Fig. 1:**
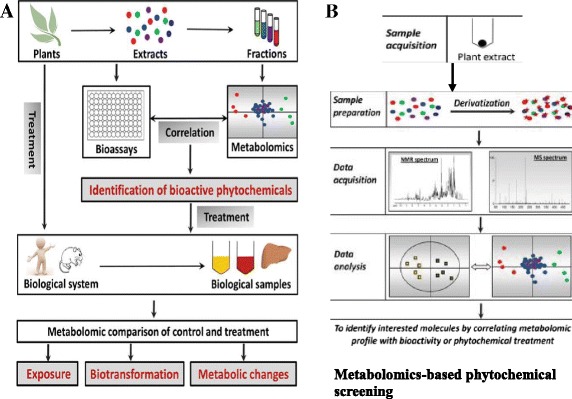
Work flow for the application of metabolomics in analysis of HMs. **a** HPLC-based activity profiling. **b** Phytochemical profiling (untargeted metabolomics) Metabolomics-based phytochemical profiling and in vitro bioassays of bioactivities may establish the correlation between specific phytochemicals and different bioactivities leading to the identification of biomarkers

Summarily, after subjecting chosen plants through the standardized extraction procedures, preliminary pharmacological assays are carried out to determine their efficacy and safety. Subject to this, the plant tissue will be subjected to derivatization and untargeted metabolomics-based phytochemical profiling. Eluted fractions will be used for relevant in vitro bioassay activities, such as multiple-parasite-stage-antiplasmodial, antioxidant, anti-inflammatory, antipyretic and antinociceptive activities. Acquired ultraviolet (UV) and MS data will be used for dereplication and tentative identification of known compounds, as well as to pinpoint potentially new compounds. The chromatogram and the activity profile will then be matched to identify active peaks. Characterization will be done by analyzing the NMR data for eluted fractions or peaks recorded off-line using microprobe technology. Metabolomics-based phytochemical profiling and in vitro bioassays may establish the correlation between specific phytochemicals and different bioactivities leading to the identification of biomarkers. Synergistic multi-target biomarkers are expected to be identified and used for in vivo validation. This data could also be used in targeted metabolomics analysis, for lead identification and eventual downstream chemical production analyses and clinical development (Fig. 1).

Although there are some standardized anti-malarial phytomedicines in current use in some endemic countries [18], the continuous and unsafe use of non-standardized plant-derived products in many malaria endemic countries obliges new approaches.

Herewith an overview of the emerging methodological landscape in the application of the multi-target phytotherapeutic concept in malaria drug discovery. This interdisciplinary approach employs tailored technology platforms coupled to miniaturized biological assays to track and characterize the anti-malarial components of herbal medicines.

By combining the modified reverse pharmacology to the systems biology approaches, the bioactivities of different phytochemicals can be assessed, in view of the identification of potential biomarkers. This will facilitate unravelling of the multi-target phytotherapeutic mechanisms and the standardization of herbal medicines as well as facilitate lead discovery for prioritization and downstream clinical analyses. Therefore, its application in malaria drug discovery could be of immense importance.

## Generation of a plant extract/compound library

A library can be defined as a collection of plant extracts/compounds stored in a standardized format and readily deliverable for screening assays. The information associated with each sample is usually stored in a database. Prior to establishing an extract library, key issues that have to be considered include the choice of plants and suitable extraction procedures to be applied to the samples.

### Choice of plants

The choice of plants as test material remains the most challenging and a determinant factor in plant-based malaria drug discovery process. There are various approaches to select plant material for this process and these include the random selection, ethnopharmacological, chemosystematic (phylogenetic), ecological and computational approaches.

The random selection of extracts and/or fractions from different plant species on the basis of their availability can be highly advantageous when samples are derived from areas of high biodiversity and malaria endemism [19]. This is because the chemical diversity of natural products can reflect the biodiversity of their source organism. Random selection of plant material can potentially result in the unexpected identification of bioactivities that would not be predicted based on indigenous or existing knowledge [20].

Ethnopharmacology is the classical approach for the choice of plants for bioassays and involves the gathering of data on the indigenous use of the plants as a basis for their selection. This usually involves the observation, description, and experimental assessment of traditionally used medicines and their effects on humans. It represents a transdisciplinary concept between the natural (botany, chemistry, biochemistry, pharmacology and pharmacognosy) and the social sciences (anthropology, archeology, history and linguistics) [12, 21]. This approach has led to the prominent discovery of some vital anti-malarials. Examples include the isolation of quinine from Cinchona species 1820 and its derivatives, chloroquine and mefloquine which replaced it in the 20^th^ century [22]. Artemisinin was isolated from *Artemisa annua* in 1971 and constitutes the core of artemisinin-based combination therapies (ACT) and its derivatives, artesunate and artemether which are widely in use currently [23]. A recently introduced plant-derived antimalarial drug is atovaquone, a synthetic naphthoquinone based on lapachol, a prenylnaphtoquinone, first isolated from *Tabebuia impetiginosa*, a South American member of the Bignoniaceae family [4]. Ethnopharmacological data is easily accessible in well-established traditional health systems such as the Traditional Chinese Medicine (TCM) and Ayurveda, which possess current documented evidence of centuries. However, this is not the case with other health systems such as the African Traditional health system, where knowledge is passed on from one generation to the other orally, and formulations in use are usually kept secret by the aging practitioners, rendering access to relevant information difficult [21, 24]. Hence, depending on the plant to be studied, there are various sources of acquiring information such as books on medical botany, review articles on medicinal plants used in different geographical regions or cultures [25] as well as computer databases [26].

The chemosystematic or phylogenetic approach is based on the chemotaxonomy and phylogeny taking into account that plant species from some genera or families are known to produce compounds and compound classes associated with a certain bioactivity or therapeutic potential [20]. The combination of phylogenetic information and ethnobotanical knowledge can be a very powerful tool in natural product drug discovery. The exploration of cross-cultural ethnomedical patterns within a phylogenetic framework can lead to high success rates [27, 28].

The ecological approach is based on the observation of interactions between organisms and their environment, considering that plant secondary metabolites possess ecological functions from which a potential therapeutic use for humans can be derived [20]. For example, compounds with antimalarial activity could be isolated from plant species eaten by chimpanzees and baboons in the wild [29].

The computational approach relies on *in silico* bioactivity predictions for constituents of certain plant species. These studies can focus on the main constituents of herbal remedies as well as on other natural compounds with anti-malarial effects directly retrieved from literature [30].

Having appropriately identified and sourced the plant(s) of choice, the next step in the value addition of medicinal plants bio-resources is the production of herbal preparations (extracts) using a variety of technologies from simple traditional technologies to advance extraction techniques, subject to the choice of assay.

### Extraction procedure

Extraction is the separation of medicinally active components (secondary metabolites) of plant tissues, using relevant solvents and standard conditions. A key aspect to the characterization of plant-derived active components is the selection of an adapted extraction protocol that would be suitable for the detection of the biomarkers of interest. This is a fundamental and critical step with important consequences for the accuracy of outcomes [31]. To increase extraction yields, plants are typically ground to a fine powder. However, when fresh material is used, it is imperative for the grinding process to be done on ice.

The aim of characterizing the bioactive components in plants is to comprehensively profile the largest possible array of low molecular weight metabolites in a given sample. In order for this to be achieved, extraction procedures with solvents that can extract compounds over a broad polarity range need to be utilized. And since there is no single solvent that has the ability to extract and dissolve all the plant metabolites at once, several solvents of increasing polarity need to be used in succession, such as hexane > ethylacetate > methanol > ethanol > water. This is the classical approach used for phytochemical screening and bio-activity guided fractionation, and it is difficult to implement in modern characterization studies known as metabolomics, because it generates many different extracts per sample that have to be analyzed separately, which usually is not compatible with the large number of samples that need to be analyzed. A dependable alternative is a combination of various solvents, such as a multi-phase solvent system composed of a mixture of solvents, such as chloroform-methanol—water, which has been proposed for such studies which are performed with pure methanol or a mixture containing it [31]. In this regard, there are various parameters to be considered in the solvent systems to be utilized, which should make it compatible with the applicable analytical technique. These include the nature of the organic and aqueous solvents, the volumes, ratios and aqueous solvent pH. For example, an aspect that needs to be considered is that lipophilic compounds do not elute from C18 reversed phase columns and certain methanol—water mixtures do not extract chlorophyll from plant aerial parts, which might be an advantage for incorporating LC analysis in MS—based metabolomics [13].

To accelerate the extraction process, additional energy needs to be applied to reduce the extraction time. This can be achieved by directly heating the system and increasing the pressure by using techniques such as the microwave-assisted extraction, supercritical fluid extraction and the mostly used pressurized (or accelerated) solvent extraction [32, 33]. These enable an efficient extraction under standardized conditions and make use of very little quantities of the plant material to be extracted, which are compatible with miniaturization of assays and the generalization of the microplate format. Moreover, extraction temperatures are usually maintained below the solvent boiling points for yield optimization and preservation of metabolite chemistry.

A review of potential anti-malarial components that have been identified from plants cover a very wide range of phytochemicals classes, which include alkaloids, terpenoids, flavonoids, coumarins, phenolics, polyacetylenes, xanthones, quinones, steroids and lignans [3, 34, 35]. Extracting each necessitates specific extraction technique, making it cumbersome and time-consuming for the investigator, especially given the fact that there might be little or no activity after extraction and isolation, as has been the case with many small molecules that have been tested for their activity. Since this reverse pharmacology – systems biology approach entails a farm - to-clinic process, whereby the ethnopharmacological knowledge and pharmacological validation is vital before HPLC-based anti-malarial profiling, solvent extracts will be dried are stored in a standardized (usually microplate) format, readily available for screening assays, biomarker and target identification, while the information associated with each sample are stored in a database.

## Choice of bioassays in in vitro efficacy and safety

In malaria drug discovery, efficacy and safety screening is a long and costly process confronted with low productivity, thereby limiting the number of new drugs. In order to improve on this, recent efforts have been dedicated to developing high throughput screening (HTS) platforms that mimic in vivo systems and provide more relevant information than biochemical assays, for the pipeline [36]. For the purposes of screening natural products, cell-based HTS methods are more relevant and typically make use of 96, 384 and 1536-well (in automated systems) microtitre plates and can be performed in a more biological relevant microenvironment. The main components are cells or parasites, culture device and detection system for quantification of cells or bioactivities. When used in screening plant extracts and other drugs, they provide representative tissue specific responses, which can be useful in efficacy and safety screening, biomarker and target identification, thereby facilitating standardization.

### In vitro antiplasmodial efficacy

In vitro screening of malaria parasite laboratory strains and field isolates for susceptibility against test compounds provides an early indication of drug efficacy, failure or possible clinical resistance. These screens are usually whole-cell based and require screening both the erythrocyte and exo-erythrocytic stages [37]. They serve first and foremost to identify active extracts before subjecting them to metabolomics analysis and secondly for the identification of potential biomarkers after metabolomics characterization of their phytochemical constituents.

In the erythrocyte stage screening, inhibition is monitored by measures of growth and proliferation of *P. falciparum* strains, usually without knowledge of the molecular targets of the compounds that are being screened [38]. HTS methods currently being used to measure *P. falciparum* in vitro drug susceptibility include the ^3^H-hypoxanthine incorporation assay [39], histidine-rich protein-2 enzyme linked immunosorbent assay (HRP-2 ELISA) [40, 41], parasite lactate dehydrogenase colorimetric assay (pLDH) [42] and the most recent SYBR Green-1 fluorescence assay [43, 44]. The titriated hypoxanthine uptake assay involves measuring parasite growth by recording levels of titriated hypoxanthine incorporation into the parasite DNA. Though accurate and reliable, its major shortcoming is the use of radioactivity, the safe disposal of which demands substantial resources. The HRP-2 method assesses parasite growth by using ELISA to measure *P. falciparum* HRP-2 and offers better result upon examining drug susceptibilities of field isolates using non-radioactive procedure. It is also more cost effective compared to the ^3^H-hypoxathine method. The pLDH assay is an indirect method that measures *P. falciparum* LDH activity as an assessment of parasitaemia by colorimetry. Although this method is more cost effective when compared to the ^3^H-hypoxanthine method, it is an ELISA method just like the HRP-2 method, which depends on the availability of specific antibodies. The recently developed SYBR Green-1 fluorescence assay is based on measuring the incorporation of the fluorescent SYBR Green-1 dye into parasite DNA. This requires a single step of DNA staining, it is less labour intensive and cost effective compared to the other methods and it is more amenable for HTS, which has become the gold standard in drug screening. This assay demonstrates the reliability of a simple, rapid, inexpensive and easy to use fluorescence-based method for drug susceptibility testing of *P. falciparum* laboratory strains and clinical isolates [45].

The exo-erythrocyte or liver stage is a very important life cycle stage with respect to the eradication programme. Recent progress has been made in the development of HTS for identification of active compounds against this stage of the parasite. There are billions of parasites present in the human blood stage infection, only a limited number of ookinetes, hypnozoites or early liver forms exists during transmission and re-infection, hence the unlikely emergence of resistance in the event of an intervention at these stages [46]. Parasites injected with the mosquito’s saliva find their way into the host liver, where they multiply asexually as exo-erythrocytic forms (EEFs) during an asymptomatic incubation period of about a week prior to emerging into the blood stream. This initiates the asexual erythrocytic cycle that is responsible for the manifestation of malaria. Contrary to the limited life span of some *Plasmodium* species, *P. ovale* and *vivax* can persist within the liver as dormant hypnozoites for several months. Their reactivation, which is usually triggered by an unknown mechanism, could see rapidly growing parasites re-populate the blood, thereby causing a pathology. Hence, the persistence of the hypnozoites represents formidable barrier to the eradication process [47].

Amongst the blood stage forms of the parasite, a subset, in response to cues that are not well understood, can differentiate into male and female gametocytes in a process that takes about 8–12 days in *P. falciparum*. These parasites metabolize the host red cell hemoglobin while progressing through five morphological distinct stages that can be identified by light microscope [48]. These immature gametocytes can last in the host for up to 55 days and mature gametocytes are the only form that can survive in the mosquito midgut, mate, undergo meiosis and give rise to next generation of parasites to be transmitted to a new host [49]. The majority of compounds active against the erythrocyte stage are inactive against the liver stage and current first line artemisin-based treatment regimens do not block transmission [50]. A current focus of anti-malarial drug discovery is to screen for small molecules and HMs that are active against both liver and gametocytes in order to block malaria transmission. The complex chemical diversity of HMs is a sure source of such scaffolds in one system.

One of the major shortcomings of developing HTS assays to evaluate liver stage activity is that parasites cannot be maintained in culture. A recently developed assay that can be used to determine the efficacy of HMs with known blood stage activity is the high content screening (HCS) on *P. yoelli* infected hepatocytes. To detect likely prophylactic activity, *P. berghei* or *P. yoelli* sporozoites are seeded unto human hepatoma cells and the infection is imaged [51] or detected enzymatically [52]. Alternatively, using a medium throughput assay, the radical potential of a test compound could be tested using the hypnozoite-forming monkey model, *P. cynolmolgi. P. cynolmolgi* sporozoites are seeded unto primary monkey hepatocytes and HCS used to determine the ratio between large, rapidly developing schizonts and dormant “small forms” (supposed hypnozoites). All parasites including hypnozoites will be eliminated by radical cure agents whereas prophylactic compounds will act on growing schizonts [53].

Though assays are available for screening compounds with transmission-blocking potential, these assays are particularly difficult because the process of gametocytogenesis is not well understood. The first gametocyte screen was conducted using alamar blue, an oxido-reduction indicator that fluoresces and changes colour in response to chemical reduction of growth medium during metabolic activity of gametocytes [54]. Based on this principle, other screens have been implemented using lactate dehydrogenase [55]. During the 12 days it takes for gametocytes to mature from the early to the late (transmission) stage, they do not divide. Consequently, methods based on the detection of inhibited parasite proliferation are limited [37]. Alternative HTS assay techniques to detect gametocyte death have been developed, most of which use overall ATP hydrolysis to measure viability. Hence the loss of this activity would signify gametocyte death [54]. It is also possible to selectively count parasites expressing a gametocyte-stage specific GFP tag by flow cytometry [56]. However, most of the assays only attempt to predict transmission-blocking activity by measuring gametocyte death. Efforts have been focused on more predictive assays aimed at measuring viability at different stages, especially the later stage [57]. Recently, Plouffe et al. [58] developed a HTS and cost effective assay technique, the Saponin-Lysis Sexual Stage Assay (SaLSSA) for identifying small molecules with transmission-blocking capacity, which clearly emphasizes substantial physiological difference between asexual and sexual parasite and provides a tool and starting point for the discovery and development of transmission-blocking drugs.

One of the limitations of the relevance of HTS antiplasmodial efficacy assays is their inability to provide information on some factors that influence the activity of substances in vivo such as absorption, distribution, metabolism, excretion and toxicity (ADME/T), which describes the disposition of bioactive compounds in in vivo systems. However, they still represent very important tools to identify and characterize active test substances [59] and this should be proceeded by toxicological screening. This is very important for the development of new drugs and for the extension of the therapeutic potential of existing molecules and herbal drugs, the first step of which is the screening on cell lines or cytotoxicity.

### Cell viability and cytotoxicity screening

Despite the growing market demand for herbal medicines, there are still concerns associated with not only their use, but their safety. Less than 10% of herbal products in the world market are truly standardized to known active components and strict quality control measures are not always diligently adhered to [60]. Therefore, cell viability and cytotoxicity assays are essential for drug screening and in vitro safety of anti-malarial drug molecules and herbal products [61]. The importance of including these assays anti-malarial plant profiling, was emphasized by van Dyk et al. [62], when they eliminated nine out of fifty-nine organic solvent extracts of South African plants that exhibited anti-malarial activity. A simple, rapid, sensitive, reliable, safe and cost-effective measurement of cells viability is the ideal test for in vitro cell proliferation and cytotoxicity and it should not interfere with the compound to be tested. These cell-based tests are performed to predict potential toxicities, using cultured cells which may be normal or transformed cells. The procedure normally involves short term exposure of cultured cells to test compounds, to detect the effects on basal or specialized cell function, prior to performing safety studies on whole organisms. It can also provide insight towards the carcinogenic or genotoxic dispositions of the test substances. The ability of a plant extract or test compound to inhibit cellular growth and viability can be ascertained as an indication of its toxicity, parameters of which include inhibition of cell proliferation, cell viability markers (metabolic and membrane), morphologic and intracellular differentiation markers [63]. Some of the factors to be considered when conducting these assays include cell culture systems, cell type compatibility with solvent system used for extraction and final concentration of dimethyl sulphoxide (DMSO) to avoid toxicity [64]. DMSO is an important aprotic solvent that can solubilize a wide variety of otherwise poorly soluble polar and nonpolar molecules, due to its amphipathic nature. This, coupled with its apparent low toxicity at concentrations <10%, has led to its ubiquitous use and widespread application [65].

Another important factor to be considered is the selection of a wide range of high quality cell types for testing including normal cells of primary origin and permanent cell lines, due to the selective toxicity displayed by some plant extracts. The advantage of using these cell lines include unlimited supply, no genetic variation, which aids reproducibility and predictive power of an outcome, as well as access to the collective knowledge gained from global research conducted on the geno- and phenotype of the cell line in question [66]. However, primary cell cultures have an advantage over most perpetual cell lines in that they are the closest in vitro representation of the in vivo cell type under scrutiny. Hence, primary hepatocytes are considered the “gold standard” used for predictive toxicology because they are often employed with the expectation that chemicals will affect or be affected by an isolated cell in the same manner that would occur in the whole organ [67].

Cell-based assays can be used to predict the mechanism of toxicity depending on the endpoint that is assessed; these include cell death/survival, adaptive/pre-lethal mechanistic reactions, including mitochondrial homeostasis, generation of reactive oxygen species (ROS), lipid peroxidation, Ca^2+^ signaling, enzyme inhibition and transporters endpoints [66].

### Toxicokinetics

Toxicokinetics deals with the prediction of toxicity due to pharmacokinetic disposition of an herb or pure compound derived from it, as a result of genetics or potential herb-drug interactions, especially given the increasing use of anti-malarial plants for therapy in endemic countries. Therefore, toxicokinetics of antimalarial components is of utmost important in evaluating the toxicity and drug interaction of anti-malarial components. Chung et al. [68] recently reported the toxicokinetics of anti-malarial artesunate in rats. This assay involves the use of human liver microsomal Cytochrome P450 isoforms and is aimed at the early identification of metabolites which are known to cause toxicological modulation in the cellular organization [69]. Cytochrome P450s are heme-containing monooxygenases located in the small intestine, liver and other tissues that play pivotal roles in the detoxification and bioactivation of diverse xenobiotics [70]. Approximately 75% of all known drugs are metabolized hepatically by mixed function oxidation reactions catalyzed by Cytochrome P450 isoforms (CYP3A4, CYP2C9, CYP2C19, CYP1A2 and CYP2D6) and they are highly subject to inhibition owing to their broad specificity for structurally diverse substrates. Modulation of Cytochrome P450 is of great significance in drug biotransformation to active or inactive forms. For drugs that are dependent on these enzymes for inactivation via conjugation to chemical polar groups prior to elimination, any herb that induces these enzymes would lead to rapid inactivation and clearance of such drugs. Herbs that inhibit enzyme activity will result to high concentrations of the drug whose inactivation relies on the enzyme activity. This could have serious implications for toxicity especially if the drug accumulated has a narrow therapeutic window. Hence, clinically significant drug-herb interactions can be observed when an herb interacts with the metabolism of a co-administered drug and either reduces its efficacy due to increased formation of an active metabolite or increases its toxicity due to reduced metabolite elimination [71]. This clinically significant effects can be predicted by analyzing in vitro metabolic data and correlating it with metabolic disposition of a test substance in vivo [72].

### In vitro therapeutic index

The in vitro therapeutic index (TI), otherwise known as the selectivity index (SI) of test compounds in malaria drug discovery research is a measure of the amount of the therapeutic agent that causes toxicity relative to that which causes the therapeutic effect (antiplasmodial activity). That is, cytotoxicity (CC_50_)/antiplasmodial activity (EC_50_ or IC_50_) [73, 74] and represents the therapeutic safety, which is the basis for selectivity of anti-malarial components for in vivo validation. In a recent study, Lima et al. [75] used SI values as a basis to select three among sixty-nine Amazonian plant extracts, for further in vivo studies. The higher the TI values for the herbal extracts or compounds derived from them, the more therapeutically safe they are considered, indicating that smaller quantities of such products will be needed to achieve high clinical efficacy with increased tolerability and safety [76]. It should be noted that although the minimum TI “cut-off” value for prioritizing samples is usually considered at SI > 4 [77], it is somewhat subjective because of the difference in susceptibilities of various cell lines and parasite strains to test compounds [78].

The effectiveness of any plant extract is dependent upon a favourable therapeutic ratio; that is the drug must kill or inhibit the parasite but have little or no toxicity to the host. The selectivity of a plant to inhibit the growth of a parasite and yet be less toxic to the host depends on differences in biochemistry between the parasite and the host. Such a plant could operate on a biochemical target in the parasite that is either absent or significantly different in the host [79].

## Characterization of the interactions between herbal extracts

Despite the use of herb-herb combinations for therapy over the past millennia, scientific evidence of their therapeutic benefits is still lacking. With the increasing interest shifting from the “one-drug-one-target” to “multi-target” or combination therapy for optimum benefits, there is momentum to explore new knowledge by tapping the past empirical experiences of herb-herb combinations. Systematic screening of combinations of some approved drugs with natural products in certain disease models has started only recently [80] but the potential of combining anti-malarial drugs and/or herbs is not yet systematically explored. There is therefore the need to characterize the interactions between anti-malarial herbal extracts and/or bioactive compounds derived from them to understand the multi-target concept in phytotherapy. These combinations may exhibit synergy at some doses, while resulting in additivity or antagonism at others. Combination dose response in in vitro antiplasmodial assays over combined dose ranges provide critical information on how different compounds act together to affect different *Plasmodium* infection outcomes [81]. The method used in combining these plant extracts is known as the fixed ratio method [59] and a modified method of combining them at equipotencies and variable potencies in antiplasmodial assays have been reported to give reproducible results [82, 83].

Briefly, active solvent extracts (say A and B) are combined in pairs at equipotency (5EC_50_A:5EC_50_B) or in variable potency (5EC_50_A:0EC_50_B to 0EC_50_A:5EC_50_B) ratios and each combination screened on resistant strains of *Plasmodium falciparum*, (such as Dd2) in an appropriate HTS assay set-up, using anti-malarial drug combinations of chloroquine/chloroquine and chloroquine/artemisinin as drug-drug interaction controls [84].

Measurements to quantify these interactions include mathematical and graphical models [85–87]. The mathematical model requires plotting the logarithm of the extract combination concentrations against activity (for example represented by the fluorescence reading) to obtain a nonlinear regression curve-fitting and a variable slope sigmoidal dose–response curve, from which inhibitory coefficients (IC_50_) of each extract in combination are determined. These are in turn used to calculate fractional inhibitory concentrations (FIC_50_A = IC_50_A in combination/IC_50_A alone) and eventually combination indices (CI = FIC_50_A + FIC_50_B), recognized as the standard measure of combination effect that indicates a greater (synergistic, CI < 1), lesser (antagonistic, CI > 1) or similar (CI = 1) effect than the expected additive effect.

Graphical models require the use of the previously calculated FIC_50_s of each extract in combination for each of the variable potency combination ratios to plot isoboles. These are plots with the line of additivity running from point (0,1) of the vertical axis to point (1,0) of the horizontal axis. Analysis of these isoboles, known as isobologram analysis, will enable the prediction of the type of interaction that exists between the plant extracts. Plant extracts in combination may produce effects that are greater than or less than their individual effects. The basis for predicting their effects in combination is based on relative potencies or dose equivalence. Therefore, a plot of the FIC_50_s of plant extracts with a constant relative potency, will produce linear additive isoboles (curves of constant effect), whereas varying potency ratios produce nonlinear additive isoboles.

**Fig. 2:**
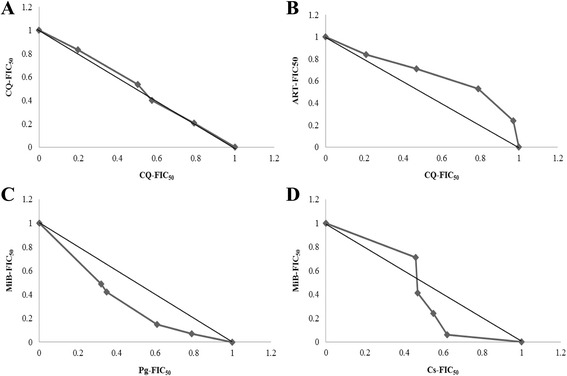
Isobolograms of the in vitro interactions between differential solvent extracts of a polyherbal product at variable potency ratios (**a**)-Additive interaction: Chloroquine/chloroquine combination (**b**)-Antagonistic interaction: Chloroquine/Artemisinin combination (**c**)-Synergistic interaction: MiB/Pg aqueous extracts combination (**d**)-Synergistic and antagonistic interactions: MiB/Cs aqueous extracts at different potency combinations [79]

Synergy or antagonism will be revealed when the plotted FIC_50_ values is situated below or above the line of additivity, respectively. An illustration of these is shown in Fig. 2, in a study carried out by Tarkang et al. [84].

Based on Chinese herbal medicine, phyto-preparations are formulated deliberately according to the following basic modes of herb-herb interactions; reinforcement, potentiation, restrain, detoxification, counteraction and toxicity. The rational of this principle suggests four mechanisms: (i) synergistic multi-target effects on enzymes, substrates, metabolites, receptors, ion channels, transport proteins and antibodies (ii) pharmacokinetic or physicochemical effects based on improved solubility, resorption rate and enhanced bioavailability (iii) antagonistic interactions with resistance mechanisms of pathogens such as *P. falciparum* (iv) elimination or neutralization of adverse effects caused by other substances present in the mixture [80, 88]. Overall, the aim remains always an effective therapy with less adverse effects.

Besides quality assurance, the proof of efficacy of phyto-preparations and the determination of their mode of action remain permanent issues for an evidence-based approach. The “omics” technologies (genomics, proteomics and metabolomics) are high throughput platforms that can provide us with these possibilities, especially for the identification of the multiple targets of identified plant constituents [88].

These in vitro analyses of the effects of polyherbals gives us an idea of effects to expect in in vivo and clinical studies. Before translating this data further in the drug discovery process, the following considerations must be made during these combination analysis: (i) an appropriate use of concepts and methods (ii) a standard reference analysis framework for characterization (iii) adaptation of the analysis to each step of the research and development process (iv) optimization of dose ratio (v) interpretation of combinations effects guided by rigorous methodology (vi) combining more than two herbs as is usually the case with polyherbals [89].

## Plant metabolomics

Metabolomics is increasingly playing a vital role in natural products drug discovery process. It aims at the comprehensive qualitative and quantitative analysis of the plant metabolome which consists of both primary and secondary metabolites, the end products of the cellular regulatory processes. This approach is based on the unbiased acquisition of MS and NMR data from specially prepared samples [13]. Application of state-of-the-art mining on this data leads to sample classification and identification of relevant biomarkers, which is very vital in lead discovery and understanding the multi-target mode of action in malaria phytotherapy. However, this method requires specific extraction (as discussed earlier) and sample preparation techniques. Since the aim is to comprehensively profile the largest possible array of low molecular weight metabolites in a given biological sample, techniques such as multiple-phase solvent systems [31] coupled to accelerated extraction processes such as microwave [90], ultrasonic [91], pressurized or accelerated liquid [92] can be applied to drastically reduce extraction times. Sample preparation usually involves the use of quality buffer and deuterated solvents that will minimize the chemical shift due to pH of the sample solution and result in good quality spectra [93].

There are various hyphenated chromatographic techniques for data acquisition such as the LC-MS, gas chromatography (GC)-MS and capillary electrophoresis (CE)-MS, as well as direct spectroscopic methods such as NMR and direct injection mass spectroscopy (DIMS). However, LC-MS is the most widely used technology in phytotherapeutic drug discovery due to its ability to detect and separate a diverse range of molecules with high sensitivity. When coupled to the NMR, it is even more versatile. The successful combination and application of these analytical tools, data mining and processing, in combination with bioassay profiling methods serve an important role in metabolomics for the purpose of dereplication in natural products-based drug discovery [17, 94].

### HPLC-based anti-malarial profiling

HPLC-based anti-malarial profiling is a highly versatile strategy to accelerate deconvolution of active herbal extracts. The principle of this approach consists of separating bioactive components of herbal extracts by analytical and semi-preparative HPLC. The first step which is a microfractionation can be performed with the bioactive sample from the generated plant library, since very little quantities of extract can be used, thereby avoiding the time-consuming recollection and extraction of samples, which at times is non-reproducible. The generated ultraviolet and MS data are recorded on-line and in combination with data searches, can be used for early dereplication and tentative identification of bioactive compounds. In parallel, fractions can be collected into microplates via a T-split of the column effluent, dried and re-dissolved in suitable solvent [95]. These will then then be assayed in vitro for their activities blood and exo-erythrocytic stage malaria parasites, using appropriate HTS assay set-ups as discussed earlier. In order to assess the full bioactivities of each extract, they will also be assayed for other activities relevant in the pathogenesis of malaria, such as antioxidant [96, 97], anti-inflammatory, antinociceptive and antipyretic [98, 99]. By matching the chromatogram and the activity profile, active peaks can be identified.

NMR data for eluted fractions and peaks are recorded off-line using microprobe NMR technology. The collected HPLC peaks can be processed in parallel and an NMR autosampler allows unattended measurement of ^1^H NMR spectra which serves as a basis for more advanced 1D and 2D analysis. If the need arises, subsequent HPLC preparative sample purification can be performed using a peak-guided strategy, since the chromatographic conditions can be easily transposed.

For a successful application of this methodology, the aspects that need to be considered include the choice of column diameter depending on the miniaturization (analytical or semi-preparative) and sensitivity of the bioassay, sample injection and column flow rates, choice of solvents for bioassays [94].

In the past decade, the application of HPLC-based anti-malarial profiling in targeted and untargeted metabolomics, for the analysis of plant extracts and HMs has been well established. Studies highlighting this potential include the quality control of various *Artemisia* species [100], correlation of antiplasmodial activities with the plants’ metabolome and efficient tracking of metabolites [101, 102]. In addition to the efficient tracking of bioactive metabolites, activity profiles can be defined, which are vital for prioritization and chemical analyses. With this information, isolation capacities can be dedicated to bioactive portions in view of facilitating the formulation of standardized and effective phytomedicines for downstream clinical development. In a recent study, Suberu et al. [103] reported the use of multivariate analysis of generated metabolomics data, to identify the association and correlation of the metabolites of artemisinin and related compounds in extracts of varieties of *Artemisia annua*. This study was aimed at determining the effects of different growth conditions, species’ origin and identification of adulterants. In another study, Allman et al. [104] reported that the metabolomics profiling of the malaria box revealed several major classes of metabolic disruption, which allows for the prediction of the mode-of-action for many of the Malaria Box compounds [104]. These results can influence the selection of appropriate drug combinations that simultaneously target multiple metabolic pathways, in future combination therapies, aimed of eliminating malaria and forestalling the expansion of drug-resistant parasites in the field.

Metabolomics has also been applied for the analysis of other biological systems such as the serum and urine of *Plasmodium*-infected hosts, in order to unveil host-pathogen interactions [105, 106]. Though still in its infancy, it enables the unveiling of novel aspects of parasite and vector biology. Furthermore, it provides insights into the biochemistry of the host, the nature of host-parasite interactions during infection, and makes it feasible for us to discover species-specific biomarkers that might correlate with various clinical manifestations of malaria.

Therefore, the metabolomics workflow and anti-malarial metabolomics profiling approach provide the malaria community with data that can be used to help prioritize the next generation of potent anti-malarial drugs for future testing in clinical trials.

Armed with the activity profile of the HMs, the next vital step is to validate these activities in animal pharmacology, as well as confirm the safety profile.

## Animal pharmacology and safety studies

In accordance with the reverse pharmacology or target-directed approach, test substances identified with good in vitro antiplasmodial activity need to be tested in vivo in suitable animal models that can provide basic pharmacological and toxicological data prior to subsequent human clinical trials [59]. Despite the fact that these in vivo assays do not necessarily predict the outcomes of clinical trials in humans because the pathogen models in animals are different from the human pathogens, animal models remain very vital in malaria phytotherapy. However, due to the reasonably high homology and similarity between mammalian genomes and physiology, and to the relatively short reproductive cycle, mouse and rat models are the most used for assessment of antiplasmodial activity of plant extracts and products derived from them [107]. Therefore, these animal models remain crucial for malaria drug evaluation and validation because they provide an integrated response encompassing efficacy, bioavailability, side effects and toxicity (ADME/T) of test substances in a whole organism and can be used for pharmacokinetic and safety studies that are pre-requisite for human clinical trials [108–110].

### Toxicity testing

While cell-based assays may be predictive of potential toxicity, use of the whole animal measures the critical toxicity of the test substance, to determine signs of toxicity as a result of gradual increase in the dose of the test substance. Many research groups are resorting to plants as a major source of new anti-malarial chemotherapeutic agents because of the extensive use of these plants for malaria therapy in African Traditional Medicine. Their use, however, does not take into consideration their safety [111]. It is therefore imperative to establish the pre-clinical safety profile of plants and their components that exhibit good anti-malarial activity in animal models. Standard guidelines have been established internationally for the care and use of animals in toxicity studies [112]. These guidelines take care of the preparation and administration of test substances, animal welfare considerations, choice of animals and regulatory requirements. The toxicity tests that are usually carried out include acute, sub-acute/sub-chronic and chronic toxicities. Exposure routes may be by oral gavage, inhalation, dermal, intraperitoneal or intramuscular, depending on the intended use and/or route of administration.

Acute toxicity testing measures relative toxicological response of an experimental organism to a single or brief exposure to a test substance [112]. This test is used to calculate lethal dose (LD_50_) [113] and observation usually lasts for fourteen days. Sub-acute (28–30 days) and sub-chronic (60–90 days) are repeated-dose daily exposure to test substances with a view of evaluating the effects on the physiological, biochemical and histopathological parameters of the organ systems of test animals. Chronic toxicity testing which is similar to sub-chronic involves the exposure of test animals over a period of six to twenty-four months or more, to reveal the effects on organ systems [114]. In addition, specialized tests may follow to reveal specific toxicities, such as reproductive, developmental, eye, skin irritancy, neuro- and geno- toxicities [115].

### In vivo antiplasmodial assays

In vivo tests are the oldest approach in the assessment of therapeutic responses and have enabled determination of the thresholds of treatment failure that are crucial for adjusting anti-malarial drug policies [116]. Test substances effective in in vitro tests are taken up for in vivo evaluation. Since *Plasmodium* species that cause human malaria are essentially unable to infect non primate animal models, in vivo evaluation of anti-malarial compounds begins with the use of rodent malaria parasites. These include *Plasmodium berghei*, *P. yoelii, P. chabaudi, P. vinckei* (blood and gametocyte stages screening) and *P. cynomolgi* (liver stage screening). In vivo models are usually employed in anti-malarial studies because they take into account the possible prodrug effect and probable involvement of the immune system in eradication of the pathogen [117]. These in vivo methods have been validated through the identification of standard drugs like mefloquine and artemisinin [118, 119] and thus remain a standard in herbal medicines drug discovery. They can be classified into primary, secondary and tertiary biological assessments [120]. In all methods, determination of percent inhibition of parasitemia is the most reliable parameter. A mean parasitemia level ≤ 90% to that of the vehicle treated animals (mock-treated control) usually indicates that the test compound is active [121].

The most widely used primary biological test is the *P. berghei* 4-Day suppressive test [122], which requires the infection of test mice with *P. berghei*, ANKA strain on day 0. 3 h post-infection and on day 1–3, the mice are treated with the test substance while using chloroquine and artemisinin as standards. On day four, blood smears from all groups of animals are prepared and stained with giemsa stain. Parasitaemia is determined from day 4 to 6 and substances identified as active are further subjected through several secondary biological assessments such as dose ranging, curative and prophylactic tests.

The dose ranging test requires a minimum of four different doses of the test substance (the “dose ranging, full 4-day test”). ED_50_ and ED_90_ values are calculated by plotting the log dose against probit activity and reflect the concentrations at which 50% and 90% of suppression of parasitaemia is achieved. This test also leads to information about the relative potency compared to an appropriate standard drug [121].

The Curative test, otherwise known as the Rane’s test, involves the daily administration of the test substance to mice or rats, 72h (D3) post established infection, for a period of five days. Parameters followed up are measurement of the reduction in blood stage parasitemia and improved survival of test animals [123]. However, in the liver stage screening, the radical cure potential of a test compound is tested using the hypnozoite-forming monkey model. Monkeys are infected with *P. cynomolgi* sporozoites followed by treatment with a compound that eliminates all blood stage parasites, followed by the test compound. The monkeys are then monitored over several months to measure reductions in the frequency of hypnozoite-caused relapses as an evaluation the potential of the test compound for radical cure [124]. The transmission-blocking activity of the test compound could also be assayed by taking advantage of the ability of gametocytes to infect mosquitoes; this can be measured by using standard membrane feeding assay, using *P. falciparum* [125], or by direct feeding of an infected mouse [126]. After feeding, the number of oocysts per mosquito midgut are counted to determine the efficacy of the test compound.

For the prophylactic test, substances with a therapeutic lead are tested for prophylactic activity by administering them for three days prior to infection and smears continually examined daily for three days, to assess suppression of blood stage parasitaemia and improved survival [127].

Tertiary biological assessments are used to monitor cross resistance and in vivo generation of drug resistance. Cross resistance monitoring employs the 4-day suppressive test in order to determine whether the same ED_50_ and ED_90_ values are observed in drug-resistant strains of rodent malaria when compared to *P. berghei* ANKA infection [121]. To determine the potential of parasites to develop resistance to a new compound in vivo, the 2% relapse method is the most widely used for most classes of compounds [128]. This entails administering a dose of the test substance that when given 1 h before infection, delays the development of 2% parasitaemia until about 7–10 days post-infection. When this parasitaemia is attained, the procedure is repeated and the time to reach 2% parasitaemia is monitored on a daily basis. The degree of resistance is expressed as a reduction in “delay time to 2%”. This method can be extended to demonstrate the stability of resistance following the removal of drug pressure and to select a partner compound that can slow the rate of development of resistance.

### Pharmacokinetics

There is evidence that the crude extracts of whole plants or mixtures thereof, which are widely used for malaria therapy, exhibit greater in vitro and/or in vivo antiplasmodial activities than isolated compounds from them, at equivalent doses. This might be as a result of synergistic interactions between their components which result in an overall positive anti-malarial effect [129]. Pharmacokinetics (PK) of these anti-malarial plants remains a major problem due to the number of plant-derived molecules that are typically present in them. This presents a substantial challenge to chemical and pharmacological evaluation, in addition to the wide concentration range of these molecules. Furthermore, the dynamic nature of the type of chemical interactions between their bioactive components and endogenous molecules have a great influence on their PK, and consequently on treatment outcomes in humans [130]. Moreover, monitoring of chemical profiles have been made more complicated due to their complex biological matrices and the lack of authenticated standards. The evolutionary considerations in interdisciplinary expertise involving ‘omics’ technologies, pharmacology, biochemistry, bioinformatics and systems modeling, offers new opportunities [94]. However, understanding their metabolic fate is a critical step toward the development of next generation of combinatorial drugs, which will maximize the synergistic and/or antagonistic effects of bioactive components and minimize their undesirable metabolic side effects [131].

The advent of comprehensive profiling technologies offers tremendous new opportunities for understanding multicomponent PK. Phytochemical profiling and metabolomics, coupled to multivariate statistical tools to generate multiparametric assessments, enable us to create a concentration-time profile of these anti-malarial plants, in a process known as a “Poly-PK” approach. This approach uses the integrated diversity of chemical composition and metabolomics profiling strategy coupled to multivariate statistical to simultaneously monitor the complex effects on the metabolic pathways of the mammalian system [131, 132]. When anti-malaria plant extracts enter mammalian bodies, the following metabolite profile changes occur over time: (i) Plant-derived compounds are absorbed into the circulation (ii) new metabolites are generated by the chemical transformation of these compounds by hepatic enzymes and gut microbes (iii) endogenous metabolites are altered in response to their intervention.

Certain PK variables such as plasma and urine concentration can be collected over time of treatment, and characterized using UHPLC-MS, while parameters such as area under the curve and the elimination half-life (t_1/2_) can be generated using PK modeling software [133]. Analysis of the various metabolomes of treated and non-treated mammals, can generate a correlation between the dynamic concentration profiles of bioactive components and the human metabolic response profile. Therefore, this approach of simultaneously monitoring the PK behaviours of multiple phytochemicals in vivo can lead to the direct elucidation of the pharmacological and molecular mechanisms underlying anti-malarial plants. This approach therefore reveals the interrelationship between xenobiotics and endobiotics, as well as the metabolic impact of these plants, using pharmacodynamics (PD) endpoints, to provide insight into the mechanisms of action and advance novel therapeutic development.

## Conclusion

Drawing from the prolonged history of phytotherapy for the clinical management of malaria, there is evidence that plant extracts exert superior effects compared to isolated phytochemicals. This is as a result of the multi-target effects of their secondary metabolites. Therefore, the success rate of developing a new drug from herbal medicinal preparations should, in theory, be higher than that from chemical synthesis. By setting quality standards to this reductionist interdisciplinary approach, potential biomarkers as well as the cellular and molecular modes of action would be unraveled. This endeavor will not only facilitate a fast and convenient discovery of efficient and standardized improved traditional medicines, but eventually lead discovery for downstream chemical analyses and prioritization. Therefore, this approach should be an asset in natural products-based drug discovery, which might shift the paradigm from the capital intensive “one-drug-to-fit-all” to the new “less investment, more drugs” model. It is believed that the former will be unsustainable in the short run, hence the need for new approaches towards the latter. Another major asset of this approach is the ethnopharmacological information providing hints for therapeutically effective plants and miniaturization, thereby reducing random and indiscriminate harvest of plants and promoting the preservation of biodiversity.

Therefore, by unifying a complete state-of-the-art reductionist and holistic techniques, the application of the multi-target phytotherapeutic concept holds great promise in widening the therapeutic arsenal against malaria.
